# High CYP2E1 activity correlates with hepatofibrogenesis induced by nitrosamines

**DOI:** 10.18632/oncotarget.22937

**Published:** 2017-12-05

**Authors:** Jie Gao, Gao-Ju Wang, Zhao Wang, Na Gao, Jing Li, Yun-Fei Zhang, Jun Zhou, Hong-Xin Zhang, Qiang Wen, Han Jin, Hai-Ling Qiao

**Affiliations:** ^1^ Institute of Clinical Pharmacology, Zhengzhou University, Zhengzhou, Henan, China; ^2^ Affiliated Cancer Hospital of Zhengzhou University, Zhengzhou, Henan, China; ^3^ Affiliated Provincial People's Hospital of Zhengzhou University, Zhengzhou, Henan, China

**Keywords:** cytochrome P450 2E1, hepatic fibrosis, causal role, prevention, diethylnitrosamine, Gerotarget

## Abstract

Hepatofibrosis, which leads to cirrhosis and eventual hepatocellular carcinoma, is a common response to chronic toxin-mediated liver injury. Nitrosamines are potent hepatotoxic agents that cause necrosis and subsequent fibrosis in the liver as a result of cytochrome P450 2E1 (CYP2E1)-dependent metabolism, which generates toxic metabolites that form adducts with nucleic acids, leading to hepatotoxicity and mutagenesis. Herein, CYP2E1 activity and content were determined in fibrotic liver tissue from patients with hepatocellular carcinoma. The relationship between CYP2E1 innate activity and hepatofibrogenesis was evaluated, the effect of inhibition of CYP2E1 activity on hepatofibrosis was determined in a Sprague-Dawley rat model of diethylnitrosamine-induced hepatofibrosis. The results demonstrated that the CYP2E1 activities in human fibrotic tissues are significantly higher than that in normal liver tissues. In rats treated with diethylnitrosamine, the livers demonstrated various degree of fibrotic changes and collagen deposition in individual rats. The Ishak score, which determines the stage of fibrosis, correlated with CYP2E1 innate activity, with greater fibrosis in rat livers with higher CYP2E1 innate activity. Inhibition of CYP2E1 during diethylnitrosamine treatment decreased hepatofibrosis and there was an inverse correlation between the degree of inhibition and the extent of hepatofibrosis. Therefore, high CYP2E1 activity is a risk factor for hepatofibrogenesis induced by nitrosamines.

## INTRODUCTION

Chronic liver diseases represent a serious global public health problem. Hepatofibrosis is a classical wound-healing response of the liver to chronic injury including infection with hepatitis virus B and C, disturbances in metabolism, and toxic insults [1, 2], ultimately leading to liver cirrhosis and hepatocellular carcinoma [3]. Because there are as yet no approved therapies, prevention and early intervention to stabilize or reverse disease progression is very important in the management of patients with hepatofibrosis [4, 5].

Nitrosamines, a large class of ubiquitous chemical carcinogens, have been found in a variety of products, including meat [6], tobacco smoke [7], and food color additives [8]. Previous studies reported that increased consumption of nitrosamines is linked to an increased risk of cancers of the gastro-intestinal tract and hepatocellular carcinoma [9–11]. Persistent fibrosis induced by chronic liver injury often precedes liver tumor formation, with hepatocellular carcinoma [12]. In humans, 80-90% of hepatocellular carcinoma patients are associated with advanced hepatic fibrosis or cirrhosis [13]. Notably, chronic and repeated insult by nitrosamines can induce fibrosis, cirrhosis, and hepatocellular carcinoma.

Cytochrome P450 2E1 (CYP2E1) is mainly expressed in the liver and plays a critical role in the metabolism of many known environmental toxicants, including nitrosamines, benzene, and carbon tetrachloride [14, 15]. As one of the more common nitrosamines, diethylnitrosamine (DEN)-induced hepatic injury is reported to be closely linked to CYP2E1's role in the metabolic activation of DEN, in which metabolites produced from DEN generate reactive electrophiles leading to cytotoxicity and carcinogenicity [14, 16]. Previous studies reported that increased CYP2E1 levels result in an enhanced activation of procarcinogens to carcinogens [17] and induction of oxidative stress [18]. In contrast, CYP2E1-knockout mice were shown to develop less oxidative stress [19] and less oxidized DNA adducts [14, 20], as compared to wild-type mice.

Our previous studies found that CYP2E1-mediated probe (chlorzoxazone) clearance *in vitro* and *in vivo* was increased two-fold in fibrotic and cirrhotic patients, while probe substrate clearance rates for nine other principal CYPs were decreased or unchanged in these patients relative to normal subjects [21, 22]. CYP2E1-mediated chlorzoxazone clearance rates varied by 20.3-fold and 13.1-fold in normal subjects and fibrotic/cirrhotic patients, respectively, resulting in about 60% of fibrotic/cirrhotic patients with higher clearance rates than normal subjects [21, 23]. Although CYP2E1-mediated chlorzoxazone clearance increased in fibrotic patients, it was not known if CYP2E1-mediated DEN metabolism might also be increased in fibrotic patients. If so, the hypothesis which proposes that higher CYP2E1 activity yields greater nitrosamine activation, resulting in greater likelihood of fibrosis, should be correct. As a consequence, we proposed that CYP2E1 is a risk factor for hepatofibrosis induced by nitrosamines.

To test the relationship between CYP2E1 activity and hepatic fibrogenesis, we characterized CYP2E1 activity in human fibrotic liver tissues and the effect of CYP2E1 innate/inhibited activity on the development of hepatofibrosis in DEN-treated rats. Our results confirm a likely causal relationship between CYP2E1 activity and hepatic fibrogenesis.

## RESULTS

### CYP2E1 activity increased significantly in patients with hepatofibrosis

**Figure 1:**
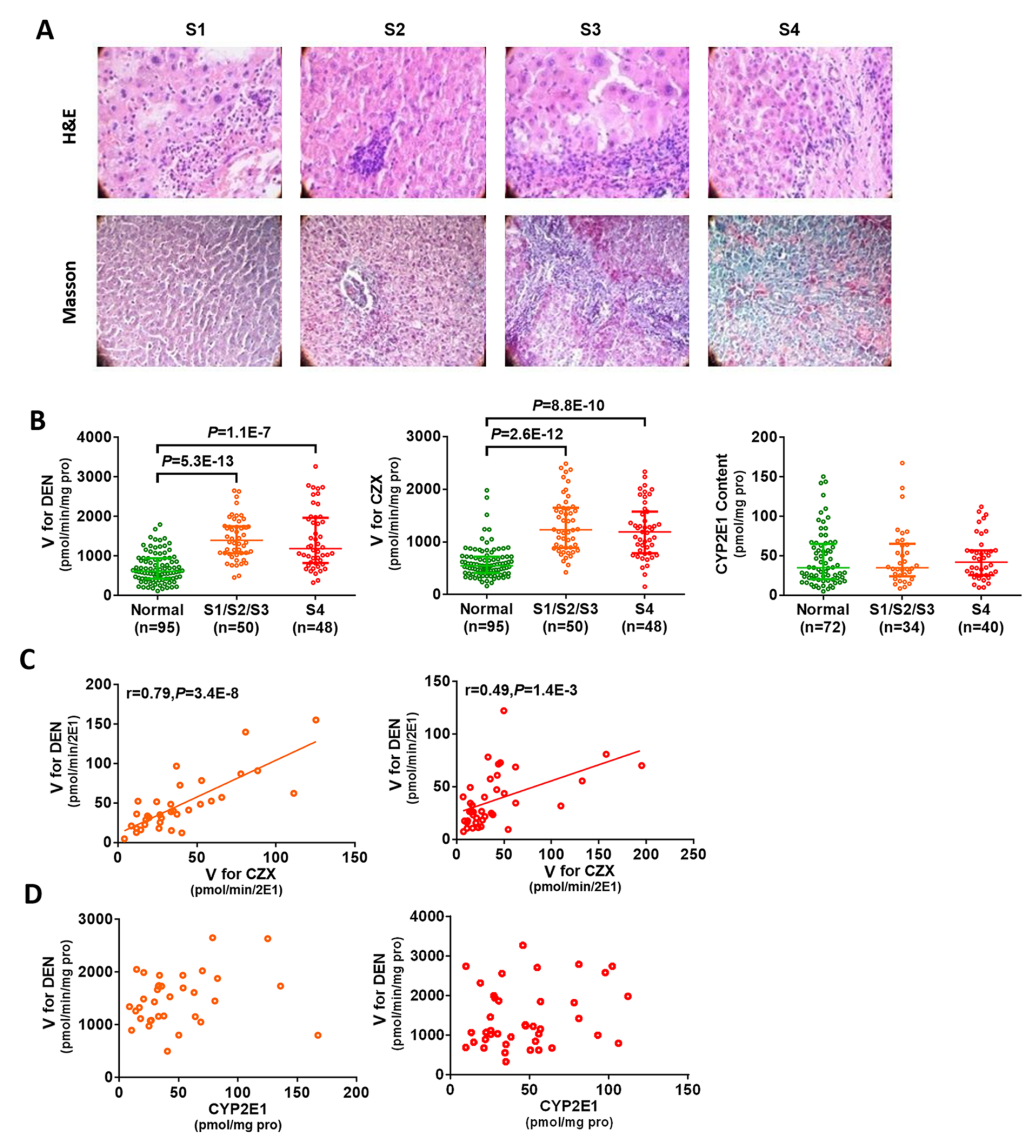
Increased CYP2E1 activity in hepatic fibrosis patients (**A**) Representative haematoxylin-eosin (H&E) and Masson's trichrome staining of liver tissue (magnification 200×). (**B**) CYP2E1 activity increased in fibrosis patients. (**C**) DEN is predominantly metabolized by CYP2E1. (**D**) There is no correlation between CYP2E1 content and CYP2E1 activity. Green circle, normal liver; orange circle, fibrotic liver; red circle, cirrhotic liver. S1: portal area fibrosis is expanded and confined in the hepatic sinus and lobule; S2: fibrosis around portal area, fibrous septums form, lobule structure maintained; S3: fibrous septums and lobule structure disordered, without cirrhosis; S4: early stage cirrhosis; CZX: chlorzoxazone, the probe substrate of CYP2E1; V: enzyme velocity; DEN: diethylnitrosamine. Data are expressed as the median with interquartile range; by Kruskal–Wallis test followed by Dunn's test, where significant correlations were determined using two-tailed Pearson correlation calculations.

Histopathological analysis confirmed 50 patients with liver fibrosis (including S1, S2, and S3) and 48 patients with liver cirrhosis (S4) (Figure 1A). The activity of CYP2E1 in the patients with hepatofibrosis was examined using the enzyme velocity (V) of CYP2E1 for chlorzoxazone and DEN. As compared to that in the normal liver tissues, CYP2E1 activity was significantly increased in fibrotic tissue. We observed a higher V for DEN in the fibrosis patients than in the normal subjects (*P*=5.3E-13), which was similar to the pattern observed for chlorzoxazone (a probe substrate for CYP2E1) metabolism (*P*=2.6E-12) (Figure 1B). In addition, there was a strong correlation in V for DEN and probe in the fibrosis patients (Figure 1C).

We next investigated the cause of increased CYP2E1 activity in fibrosis tissues. We determined that CYP2E1 content was not very likely to be increased in the fibrosis tissues, as Enzyme-Linked Immuno-Sorbent Assay detected equal amounts of CYP2E1 from equal amounts of normal and fibrotic liver tissue (Figure 1D). Furthermore, we found that there was no correlation between CYP2E1 activity and content (Figure 1D).

Taken together, these data suggest that the CYP2E1 activity might be a promising biomarker for hepatofibrosis, although the underlying mechanism of increased CYP2E1 activity in fibrosis remains to be established.

### Higher CYP2E1 innate activity is associated with increased risk of hepatofibrosis

**Figure 2:**
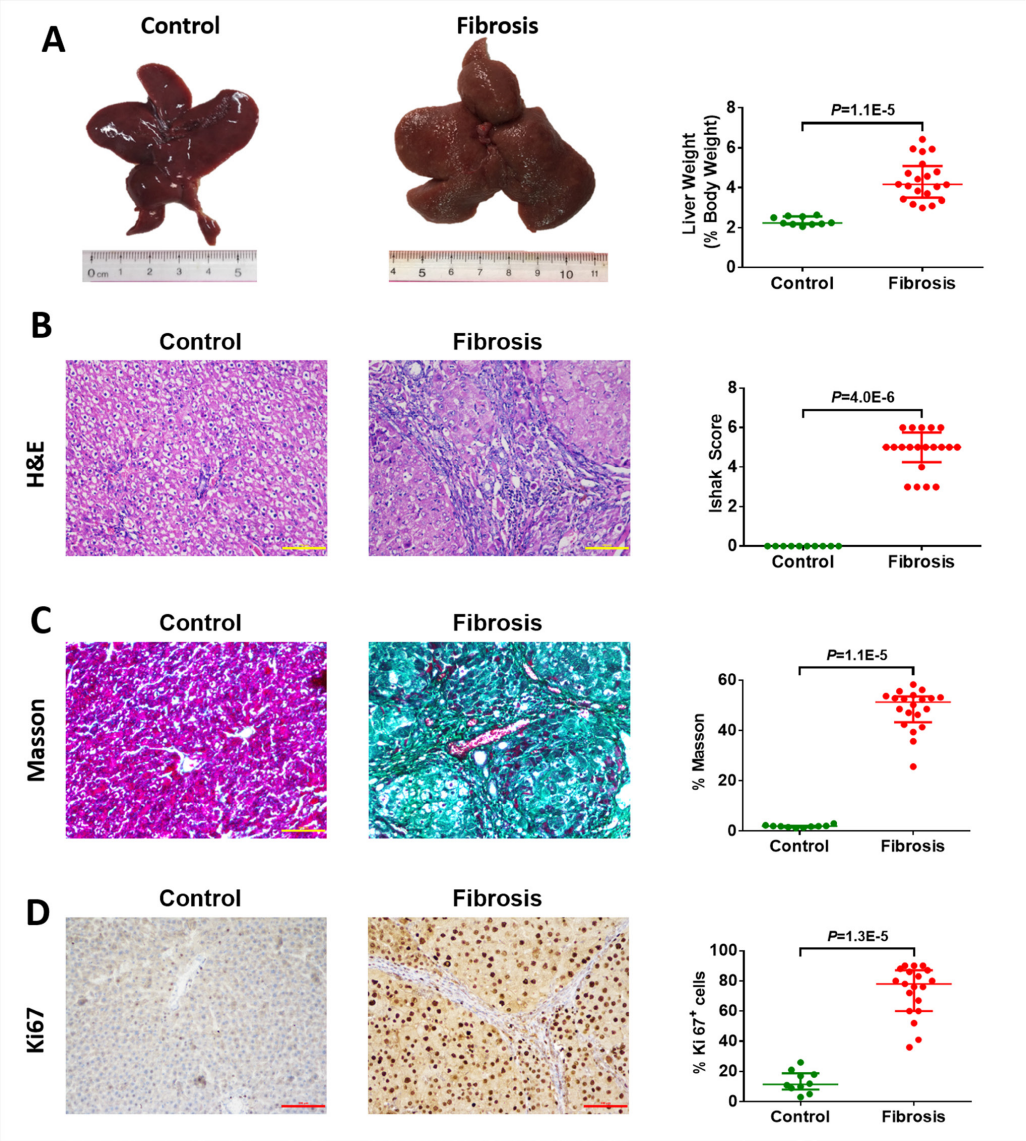
Macroscopic and microscopic findings on control liver tissue (n=10) and diethylnitrosamine (DEN)-induced hepatofibrotic liver tissue (n=20) in male rats (**A**) Representative rat livers at week 12. Representative haematoxylin-eosin (H&E) (**B**), trichrome staining (**C**), and Ki67 staining (**D**) of liver tissue (magnification 200×) at week 12. Data are expressed as the median with interquartile range; by Mann-Whitney U test.

As CYP2E1 activity is significantly increased in fibrosis patients, we speculated that CYP2E1 is a risk factor for liver injury and fibrosis induced by nitrosamines. We investigated the relationship of CYP2E1 activity to hepatofibrosis in experimental models *in vivo*. Macroscopically, the liver weight (% body weight) of hepatofibrotic rats was significantly increased at week 12 compared with control rats (Figure 2A). Histopathologic examination revealed centrilobular atypia and multiple larger dysplastic foci but the degree of hepatofibrosis was different in individual rats (Figure 2B). Meanwhile, there was notable collagen deposition demonstrated by Masson's trichrome staining and marked hepatocyte proliferation as assessed by immunohistochemical staining for Ki-67 in the hepatic fibrosis rat models (Figure 2C and 2D).

**Figure 3:**
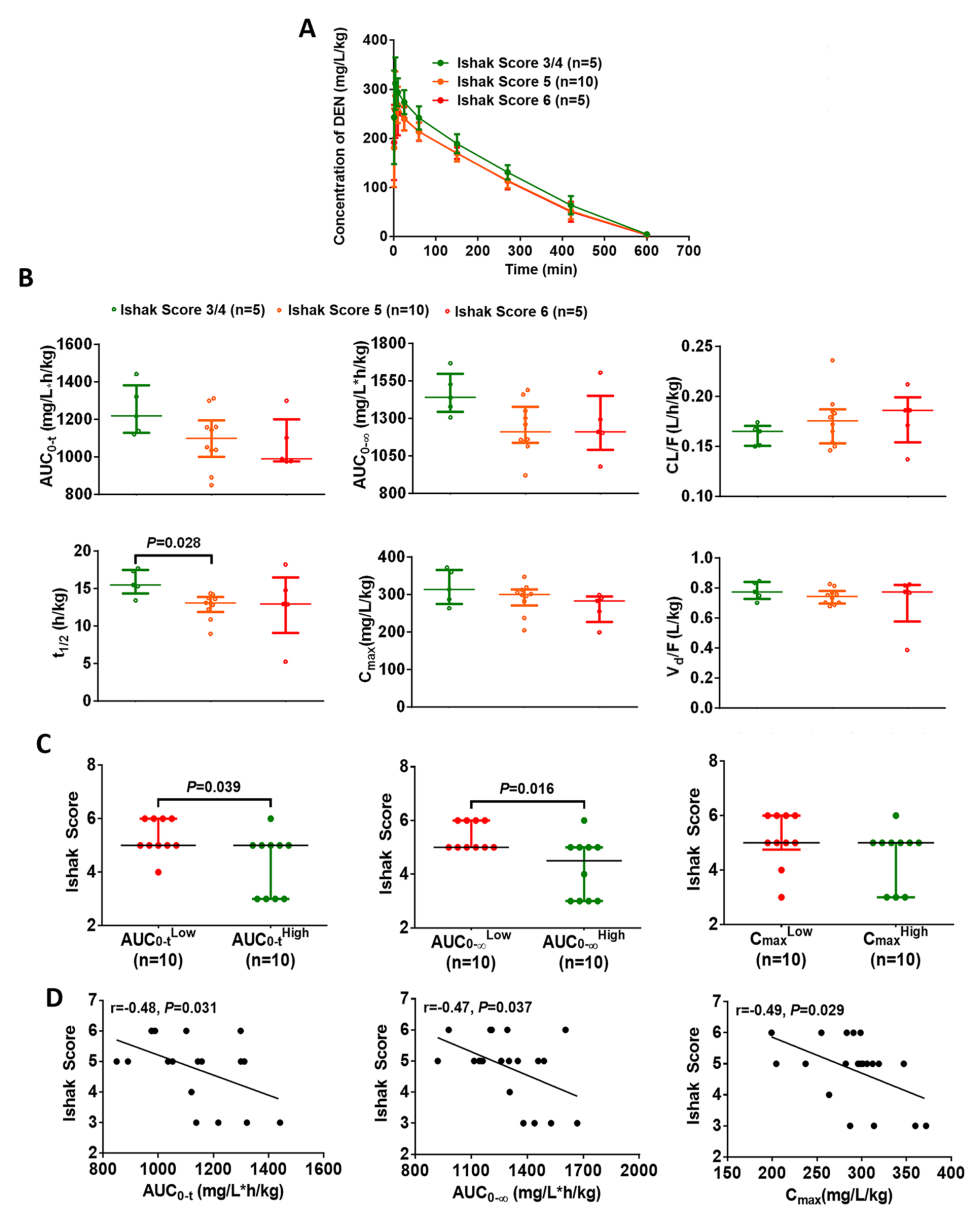
Elevated CYP2E1 innate activity is associated with increased risk of hepatofibrosis (**A**) Time course of the plasma concentration of diethylnitrosamine (DEN) (50 mg/kg, i.p.) in the DEN-induced hepatic fibrosis models. Data are expressed as the median with interquartile range. (**B**) Toxicokinetic parameters of DEN (50 mg/kg, i.p.) in rats with different degree of fibrosis. Data are expressed as the median with interquartile range; Kruskal–Wallis test followed by Dunn's test was performed. (**C**) The effect of CYP2E1 innate activity on the degree of hepatofibrosis. Data are expressed as the median with interquartile range; Mann-Whitney U test was used. (**D**) The correlation between the toxicokinetic parameters of diethylnitrosamine metabolism and the stage of fibrosis (n=20). Pearson's correlation test was used.

Beginning with establishment of the hepatofibrosis model (at week 0), the CYP2E1 innate activity in each rat was estimated by measuring the plasma concentration of DEN at different time points, with determination of toxicokinetic parameters (area under curve, AUC; apparent clearance, CL/F; half-life, t_1/2_; maximum concentration, C_max_; apparent volume of distribution, V_d_/F) (Figure 3A and 3B). We found that the t_1/2_ of mildly fibrotic rats (Ishak Score = 3/4) was observably longer than that of moderately fibrotic rats (Ishak Score = 5) (Figure 3B). Using the median value as the cutoff, we found that a low AUC of DEN in hepatic fibrosis rat models was associated with severe fibrosis (Figure 3C). Further, AUC_0-t_, AUC _0-∞_, and C_max_ both were inversely correlation with the degree of hepatofibrosis (Figure 3D). These data demonstrate that the higher CYP2E1 innate activity should be a risk factor for hepatofibrosis.

### Inhibition of CYP2E1 activity prevents DEN-induced rat hepatofibrosis

**Figure 4:**
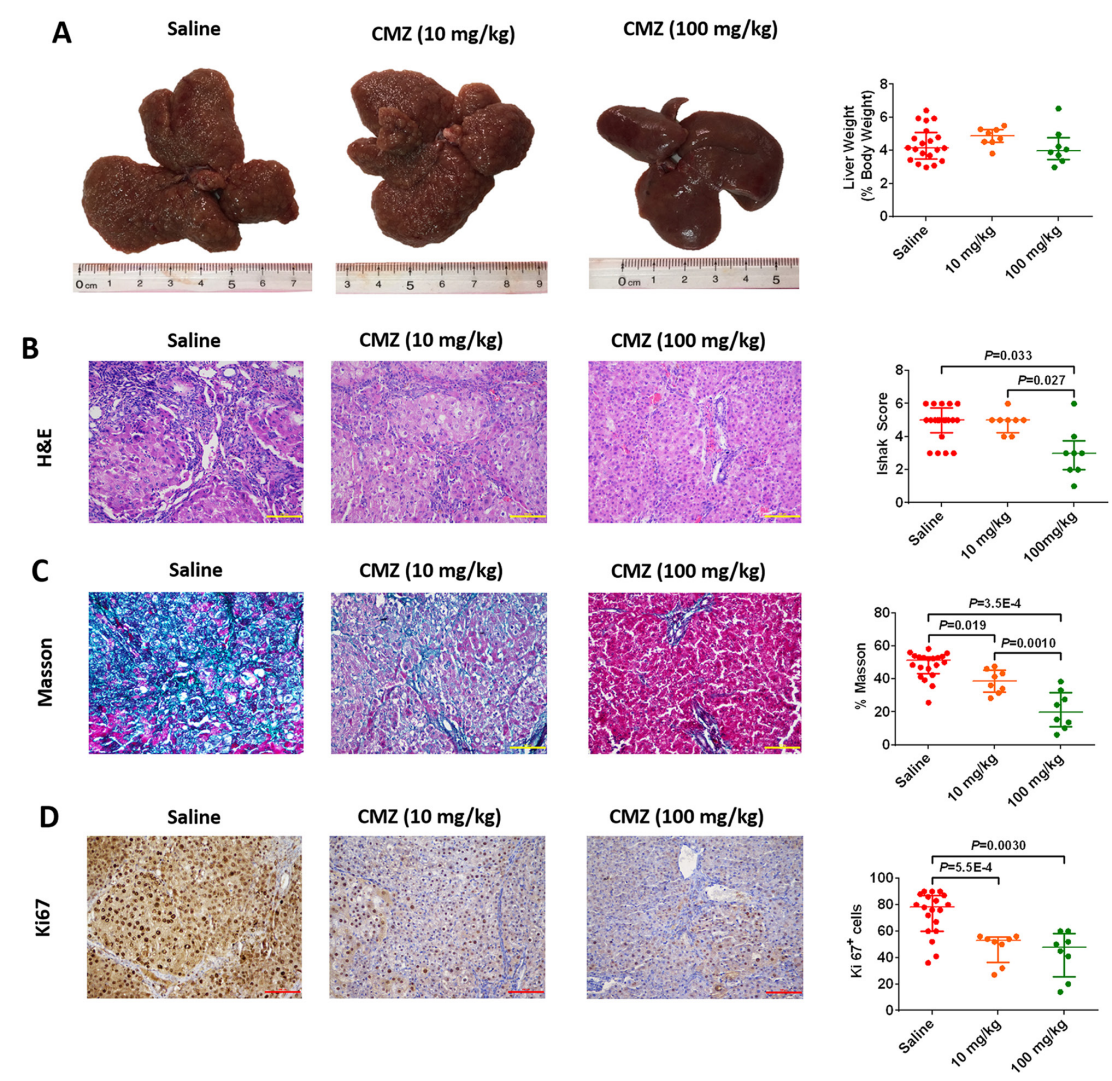
Chlormethiazole (CMZ) prevented diethylnitrosamine (DEN)-induced liver fibrosis Saline (n=20), 10 mg/kg CMZ (n=8), 100 mg/kg CMZ (n=8). (**A**) Representative rat livers at week 12. Representative haematoxylin-eosin (H&E) (**B**), trichrome staining (**C**), and Ki67 staining (**D**) of liver tissue (magnification 200×) at week 12. Data are expressed as the median with interquartile range; by Kruskal–Wallis test followed by Dunn's test.

Having established that higher CYP2E1 innate activity correlates with greater fibrosis, we used chlormethiazole, a CYP2E1 inhibitor, to examine the role of CYP2E1 in DEN-induced hepatofibrosis. To further determine whether CYP2E1 is a risk factor for hepatofibrogenesis, saline or chlormethiazole (10 mg/kg or 100 mg/kg) was administered by intraperitoneal injection before injecting DEN. In this intervention experiment liver weight (% body weight) did not differ between these groups (Figure 4A). Chlormethiazole caused a substantial and significant reduction in fibrosis as measured by either Sirius red staining or collagen staining (Figure 4B and 4C), and decreased hepatocellular proliferation as measured by immunohistochemical staining for Ki-67 (Figure 4D). These results suggest that chlormethiazole could reduce or prevent rat hepatofibrosis induced by DEN.

**Figure 5:**
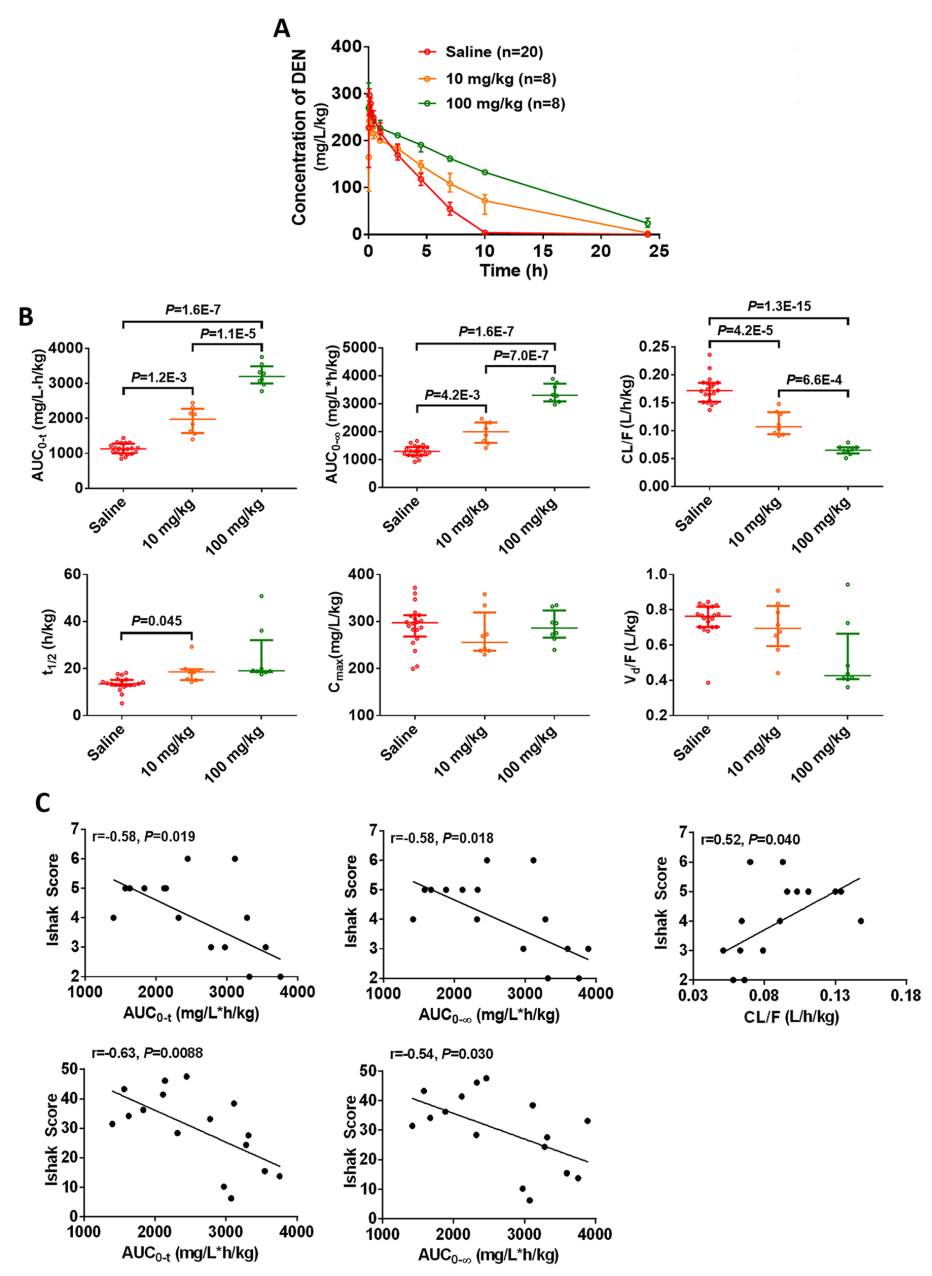
CYP2E1 inhibition by chlormethiazole (CMZ) is beneficial for the prevention of diethylnitrosamine (DEN)-induced liver fibrosis Saline (n=20), 10 mg/kg CMZ (n=8), 100 mg/kg CMZ (n=8). (**A**) Time course of the plasma concentration of DEN at week 0. Data are expressed as the median with interquartile range. (**B**) Toxicokinetic parameters of DEN in liver fibrosis models treated with saline or chlormethiazole (10 mg/kg or 100 mg/kg). Rats received DEN (50 mg/kg) via intraperitoneal injection together with CMZ, DEN was given immediately after the injection of CMZ. Data are expressed as the median with interquartile range; by Kruskal–Wallis test followed by Dunn's test. (**C**) The correlation between inhibited toxicokinetic parameters of DEN and fibrosis degree (n=16).

Further, we confirmed the involvement of CYP2E1 in the chlormethiazole-induced reduction of hepatofibrosis. After 2.5 hours, rats receiving 10 mg/kg and 100 mg/kg chlormethiazole had higher concentration of DEN in plasma than those administered saline (Figure 5A). The AUC_0-t_ and AUC_0-∞_ were significantly increased, and CL/F was markedly decreased in a dose-dependent pattern (Figure 5B). The above data confirm that chlormethiazole inhibits CYP2E1 activity. We next evaluated the relationship between CYP2E1 activity in the presence of chlormethiazole treatment and the degree of fibrosis (Figure 5C). These data demonstrate that inhibition of CYP2E1 activity prevents or reverses DEN-induced rat hepatofibrosis.

In summary, the results obtained from human and animal studies support the hypothesis that CYP2E1 is a risk factor for hepatofibrogenesis induced by nitrosamines.

## DISCUSSION

This study suggests that CYP2E1 is a risk factor for nitrosamine-induced hepatofibrosis. In fibrotic liver tissues, CYP2E1 activity was increased while CYP2E1 content was not changed, as compared with normal human liver tissues. In addition, the higher CYP2E1 innate activity exerted a pro-fibrogenic effect, perhaps by enhancing the activation and subsequent toxicity of DEN; inhibition of CYP2E1 prevented hepatofibrosis in the nitrosamine-induced model of hepatofibrosis.

Hepatofibrosis is a life-threatening disease [24] and there are few effective therapies despite the current use of some small molecules (e.g. atrasentan, nilotinib, GKT-137831, GFT-505) in the clinic [4]. Chronic liver injury from many causes, including persistent viral infection, alcohol, and oncogenic agents commonly results in hepatofibrosis, hepatocirrhosis, and ultimately, hepatocellular carcinoma [3]. Repeated exposure to nitrosamines contained in the typical diet in most countries can trigger chronic liver injury [9, 25]. Therefore, early prevention and intervention in hepatofibrosis may play an important role in preventing hepatocarcinogenesis.

DEN is a well-established hepatotoxin and hepatocarcinogen, leading to necrosis and fibrosis in the liver. It is one of the most common hepatotoxic agents used in experimental models [26]. DEN-induced hepatofibrosis in rats is similar to the progression of fibrosis in humans [26, 27]. In addition, DEN has been widely found in various products, including processed meats [28], pickles [29], tobacco smoke [30], and food color additives [8]. For the above reasons DEN-induced hepatofibrosis was utilized in this study to determine if CYP2E1 is a risk factor for nitrosamine-induced hepatofibrosis.

It long has been known that CYP2E1 could convert many toxicologically important substrates (ethanol, carbon tetrachloride, and nitrosamines) to reactive intermediates, eliciting organ damage and tumorigenesis [14, 15]. DEN-induced hepatic injury is reported to be closely related to CYP2E1 mainly expressed in the liver [31]. Although some previous studies suggested that CYP2E1 plays a crucial role in hepatic injury, CYP2E1 content rather than CYP2E1 activity was typically measured and linked to liver injury [32, 33]. In the present study, we found that there is no correlation between CYP2E1 content and its activity.

Because DEN is mainly metabolized by CYP2E1 *in vivo* [7, 34], we determined CYP2E1 activity in our rat hepatofibrosis model by measuring the toxicokinetic parameters (AUC, CL/F, t_1/2_, C_max_, and V_d_/F) of DEN in saline- and chlormethiazole-treated rats. AUC reflects the amount of DEN entering the blood circulation, CL/F is the volume of plasma containing DEN that is removed from the body per unit time, t_1/2_ refers to the time required to eliminate half of DEN in the body, C_max_ is the maximum concentration of DEN in the body, V_d_/F is the ratio of DEN dose to blood concentration of DEN when DEN reaches a dynamic balance *in vivo*. These toxicokinetic parameters represent the activity of CYP2E1 towards DEN. We further analyzed the association between the CYP2E1 activity and the degree of fibrosis. Our results suggest that rats with higher CYP2E1 activity exhibited more severe fibrosis, indicating that inhibition of CYP2E1 activity should be able to moderate hepatic fibrosis.

Indeed, after CYP2E1 inhibition, we observed less fibronectin formation, collagen deposition, and hepatocyte proliferation, as compared with livers from animals not treated with chlormethiazole, suggesting that CYP2E1 inhibition might have a role in protecting hepatocytes from DEN toxicity. This provides support for the possibility of CYP2E1 serving as a risk factor for hepatofibrosis. However, the details of the mechanisms by which CYP2E1 inhibition reduces liver injury and fibrogenesis remain to be worked out.

Chlormethiazole could significantly inhibits the metabolism of DEN and alleviate hepatic fibrosis by reducing the production of hepatotoxic agents, but does not prevent hepatic fibrosis entirely. The reason behind this could be that the dose of chlormethiazole and the intermittent dosing schedule used in the study cannot completely inhibit CYP2E1 activity. Increasing the dose of this inhibitor is limited by the toxicity of chlormethiazole itself. As a consequence, more safe and efficient inhibitors of CYP2E1 will need to be developed to pursue this line of investigation.

This is the first direct demonstration that the activity of CYP2E1 for DEN was significantly increased in human fibrotic liver tissues, that higher CYP2E1 innate activity correlated with hepatofibrosis, and that inhibition of CYP2E1 activity limited liver fibrosis in response to DEN. The present study supports the value of CYP2E1 inhibitors in the prevention hepatofibrogenesis in human and animal models. As there are few effective therapeutic strategies for preventing and treating hepatic fibrosis, our results should have immediate and important clinical implications for preventing hepatic fibrosis.

## MATERIALS AND METHODS

### Patients and samples

We obtained surrounding nontumor hepatic tissues from 98 hepatocellular carcinoma patients who had undergone radical resection between 2013 and 2014, and normal liver tissues (n=95) from subjects with liver hemangioma, metastatic carcinoma, cholelithiasis, or gallbladder cancer between 2012 and 2013 at Affiliated Provincial People's Hospital of Zhengzhou University, and the Affiliated Cancer Hospital of Zhengzhou University. All patients received a liver function test, histopathological analysis, and imaging (ultrasonography or computer tomography). Liver specimens were stored in liquid nitrogen within 30 min of resection and until use. The Medical Ethics Committee of Zhengzhou University, Zhengzhou, China approved the tissue collection and *in vitro* metabolism studies, and patients provided written informed consent for the use of all surgically-removed tissue specimens.

### Experimental animals

Male Sprague–Dawley rats (160±20 g, 5-week-old, No.11400700149357) were housed in a virus-free facility and maintained in a standard temperature- and light-controlled animal facility and had free access to standard laboratory chow and water. Animals were supplied by Beijing Vital River Laboratory Animal Technology Co., Ltd. Experiments were initiated after acclimation to the above conditions for 1 week. All animal experiments were undertaken in accordance with the National Institute of Health Guide for the Care and Use of Laboratory Animals, with the approval of the Medical Ethics Committee of Zhengzhou University, Zhengzhou, China.

### CYP2E1 content

CYP2E1 content was estimated by Enzyme-Linked Immuno-Sorbent Assay. Briefly, about 30 mg liver tissue stored in liquid nitrogen was defrosted on ice and weighed. Phosphate buffer (10 mM, pH 7.0) was added 1:20 (W:V) and the tissue homogenized at 4°C, followed by centrifugation at 6000 g for 10 min, recovering the supernatant fraction. A 10 μl aliquot of supernatant was diluted 40-fold for analysis, performed according to standard procedures provided by the manufacturer (Wuxi Donglin Sci & Tech Development Co., Ltd.).

### CYP2E1 activity

Human liver microsomes were prepared using the hypothermal differential centrifugation method [35]. Chlorzoxazone was purchased from the National Institute for the Food and Drug Control (China), 6-hydroxychlorzoxazone was from Sigma-Aldrich (St. Louis, MO, USA). DEN was from TCI Shanghai. Aldehyde and 2,4-dinitrophenylhydrazine were from Aladdin^®^.

The CYP2E1 activity of normal subjects and fibrotic/cirrhotic patients was determined at a single concentration in individual assays by incubation of microsomal protein (0.3 mg), phosphate buffer (100 mM, pH 7.4), and substrate (500 μM chlorzoxazone or 160 mM DEN). The mixture was incubated for 5 min at 37°C and the reaction initiated by adding 1 mM NADPH. Each reaction was terminated after the specified incubation period by adding 1 ml ethyl acetate (chlorzoxazone) or 10 μl perchloric acid (DEN). The 6-hydroxychlorzoxazone was extracted using 1 ml ethyl acetate, concentrated to dryness under nitrogen, and dissolved in mobile phase for analysis by high-pressure liquid chromatography (HPLC) using a Diamonsil C_18_ column (5 μm, 4.6 mm×200 mm; Dikma Technologies, China). Samples were eluted with methanol/water (55:45; v/v) at a flow rate of 1.0 mL/min. The eluates were monitored by an ultraviolet detector at 287 nm. For aldehyde determinations, 100 μl aliquots of supernatant after 10 μl perchloric acid precipitation were added to tubes containing 50 μl of 1 mg/mL 2,4-dinitrophenylhydrazine in 6 M HCl , 2 mL isooctane, and 2 mL of 3 M sodium acetate. The tubes were vigorously shaken for 20 min at 60°C. A 1.6 mL sample of the isooctane layer was dried under nitrogen and dissolved in 200 μl mobile phase for analysis by HPLC using a Diamonsil C_18_ column. Samples were eluted with acetonitrile/water (75:25; v/v) at a flow rate of 1.0 mL/min. The eluates were monitored by an ultraviolet detector at 356 nm.

The CYP2E1 innate activity of each rat was determined at week 0 by measuring toxicokinetic parameters (area under curve, AUC; apparent clearance, CL/F; half-life, t_1/2_; maximum concentration, C_max_; apparent volume of distribution, V_d_/F) of DEN (50 mg/kg) which was administered by intraperitoneal injection to healthy male rats. Blood samples (0.5 mL) were collected before DEN dosing and at 1, 4, 10, 25, 60, 150, 270, 420, 600, and 1440 min after DEN dosing by orbital bleeding into heparinised tubes. Plasma was obtained by centrifugation at 4500 rpm for 10 min at 4°C. Plasma protein was precipitated with 10 μl perchloric acid with vortex-mixing for 60 s followed by centrifugation at 4°C and 12,000 rpm for 10 min. A 10 μl sample of the supernatant was analyzed by HPLC using a Diamonsil C_18_ column. Samples were eluted with methanol/water (50:50; v/v) at a flow rate of 1.0 mL/min. The eluates were monitored by an ultraviolet detector at 240 nm.

The CYP2E1 inhibited activity in the presence of an inhibitor (chlormethiazole) was determined by measuring the toxicokinetic parameters of DEN metabolism (50 mg/kg) injected intraperitoneally immediately after chlormethiazole (10 mg/kg or 100 mg/kg) to healthy male rats at week 0. Other experiments were as described above.

### DEN-induced rat hepatofibrosis models

Six-week-old male Sprague–Dawley rats were injected intraperitoneally with DEN (50 mg/kg) twice a week for the first four weeks and once a week over the next eight weeks. Saline was used as a vehicle control and was injected into “control” rats at the time of DEN administration. Chlormethiazole was used as a CYP2E1 inhibitor in the intervention experiment, in which DEN (50 mg/kg) was injected immediately after chlormethiazole (10 mg/kg or 100 mg/kg) or an equal volume of saline administration. At 12 weeks the animals were killed and the liver was photographed, sectioned and fixed in 10% formalin. The remaining portions of the liver were instantly frozen and stored in liquid nitrogen until use.

### Histology and immunohistochemistry

Formalin-fixed samples were embedded in paraffin, cut into 5 μm-thick sections and stained with hematoxylin and eosin and Masson's trichrome according to standard procedures. Trichrome-stained sections were analyzed to score fibrosis staging according to the method of Ishak [36]. Additional sections were stained with antibodies specific for Ki67 (Abcam, ab29). After staining for Ki67, positive cells were morphometrically quantified with image processing software (Image-pro-plus)

### Statistical analyses

All data were analyzed using SPSS software (version 17.0). For data sets with skewed distributions, nonparametric statistical analysis was performed using the Mann-Whitney U test for two groups; the Kruskal–Wallis test followed by Dunn's test was utilized for multiple comparisons. In addition, significant correlations were determined using two-tailed Pearson correlation calculations. A *P* value of 0.05 or less was considered to indicate statistical significance. All graphs were generated using the Adobe Photoshop CC 2014, PowerPoint 2013 and GraphPad Prism version 6.0 software package.

## Abbreviations

CYP2E1: cytochrome P450 2E1
DEN: diethylnitrosamin
HPLC: high-pressure liquid chromatography
AUC: area under curve
CL/F: apparent clearance
t_1/2_: half-life
C_max_: maximum concentration
V_d_/F: apparent volume of distribution
V: enzyme velocity.

## References

[1] Ding BS, Cao Z, Lis R, Nolan DJ, Guo P, Simons M, Penfold ME, Shido K, Rabbany SY, Rafii S (2014). Divergent angiocrine signals from vascular niche balance liver regeneration and fibrosis. Nature.

[2] Gurtner GC, Werner S, Barrandon Y, Longaker MT (2008). Wound repair and regeneration. Nature.

[3] European Association for the Study of the Liver; European Organisation for Research and Treatment of Cancer (2012). EASL-EORTC clinical practice guidelines: management of hepatocellular carcinoma. J Hepatol.

[4] Nanthakumar CB, Hatley RJ, Lemma S, Gauldie J, Marshall RP, Macdonald SJ (2015). Dissecting fibrosis: therapeutic insights from the small-molecule toolbox. Nat Rev Drug Discov.

[5] Ge PS, Runyon BA (2016). Treatment of Patients with Cirrhosis. N Engl J Med.

[6] Herrmann SS, Granby K, Duedahl-Olesen L (2015). Formation and mitigation of N-nitrosamines in nitrite preserved cooked sausages. Food Chem.

[7] Hengstler JG, Bogdanffy MS, Bolt HM, Oesch F (2003). Challenging dogma: thresholds for genotoxic carcinogens? The case of vinyl acetate. Annu Rev Pharmacol Toxicol.

[8] Tsuda S, Murakami M, Matsusaka N, Kano K, Taniguchi K, Sasaki YF (2001). DNA damage induced by red food dyes orally administered to pregnant and male mice. Toxicol Sci.

[9] Song P, Wu L, Guan W (2015). Dietary Nitrates, Nitrites, and Nitrosamines Intake and the Risk of Gastric Cancer: A Meta-Analysis. Nutrients.

[10] Jakszyn P, Agudo A, Berenguer A, Ibanez R, Amiano P, Pera G, Ardanaz E, Barricarte A, Chirlaque MD, Dorronsoro M, Larranaga N, Martinez C, Navarro C (2006). Intake and food sources of nitrites and N-nitrosodimethylamine in Spain. Public Health Nutr.

[11] Knekt P, Jarvinen R, Dich J, Hakulinen T (1999). Risk of colorectal and other gastro-intestinal cancers after exposure to nitrate, nitrite and N-nitroso compounds: a follow-up study. Int J Cancer.

[12] Elsharkawy AM, Mann DA (2007). Nuclear factor-kappaB and the hepatic inflammation-fibrosis-cancer axis. Hepatology.

[13] El-Serag HB (2011). Hepatocellular carcinoma. N Engl J Med.

[14] Kang JS, Wanibuchi H, Morimura K, Gonzalez FJ, Fukushima S (2007). Role of CYP2E1 in diethylnitrosamine-induced hepatocarcinogenesis in vivo. Cancer Res.

[15] Gonzalez FJ (2005). Role of cytochromes P450 in chemical toxicity and oxidative stress: studies with CYP2E1. Mutat Res.

[16] Glauert HP, Calfee-Mason K, Stemm DN, Tharappel JC, Spear BT (2010). Dietary antioxidants in the prevention of hepatocarcinogenesis: a review. Mol Nutr Food Res.

[17] Dinis-Oliveira RJ (2016). Oxidative and Non-Oxidative Metabolomics of Ethanol. Curr Drug Metab.

[18] Seitz HK, Stickel F (2007). Molecular mechanisms of alcohol-mediated carcinogenesis. Nat Rev Cancer.

[19] Lu Y, Wu D, Wang X, Ward SC, Cederbaum AI (2010). Chronic alcohol-induced liver injury and oxidant stress are decreased in cytochrome P4502E1 knockout mice and restored in humanized cytochrome P4502E1 knock-in mice. Free Radic Biol Med.

[20] Bradford BU, Kono H, Isayama F, Kosyk O, Wheeler MD, Akiyama TE, Bleye L, Krausz KW, Gonzalez FJ, Koop DR, Rusyn I (2005). Cytochrome P450 CYP2E1, but not nicotinamide adenine dinucleotide phosphate oxidase, is required for ethanol-induced oxidative DNA damage in rodent liver. Hepatology.

[21] Zhou J, Wen Q, Li SF, Zhang YF, Gao N, Tian X, Fang Y, Gao J, Cui MZ, He XP, Jia LJ, Jin H, Qiao HL (2016). Significant change of cytochrome P450s activities in patients with hepatocellular carcinoma. Oncotarget.

[22] Gao J, Zhou J, He XP, Zhang YF, Gao N, Tian X, Fang Y, Wen Q, Jia LJ, Jin H, Qiao HL (2016). Changes in cytochrome P450s-mediated drug clearance in patients with hepatocellular carcinoma in vitro and in vivo: a bottom-up approach. Oncotarget.

[23] Gao N, Tian X, Fang Y, Zhou J, Zhang H, Wen Q, Jia L, Gao J, Sun B, Wei J, Zhang Y, Cui M, Qiao H (2016). Gene polymorphisms and contents of cytochrome P450s have only limited effects on metabolic activities in human liver microsomes. Eur J Pharm Sci.

[24] Satoh T, Nakagawa K, Sugihara F, Kuwahara R, Ashihara M, Yamane F, Minowa Y, Fukushima K, Ebina I, Yoshioka Y, Kumanogoh A, Akira S (2017). Identification of an atypical monocyte and committed progenitor involved in fibrosis. Nature.

[25] Chappell G, Kutanzi K, Uehara T, Tryndyak V, Hong HH, Hoenerhoff M, Beland FA, Rusyn I, Pogribny IP (2014). Genetic and epigenetic changes in fibrosis-associated hepatocarcinogenesis in mice. Int J Cancer.

[26] Starkel P, Leclercq IA (2011). Animal models for the study of hepatic fibrosis. Best Pract Res Clin Gastroenterol.

[27] Schiffer E, Housset C, Cacheux W, Wendum D, Desbois-Mouthon C, Rey C, Clergue F, Poupon R, Barbu V, Rosmorduc O (2005). Gefitinib, an EGFR inhibitor, prevents hepatocellular carcinoma development in the rat liver with cirrhosis. Hepatology.

[28] De Mey E, De Klerck K, De Maere H, Dewulf L, Derdelinckx G, Peeters MC, Fraeye I, Vander Heyden Y, Paelinck H (2014). The occurrence of N-nitrosamines, residual nitrite and biogenic amines in commercial dry fermented sausages and evaluation of their occasional relation. Meat Sci.

[29] Chu JJ, Xu YC, Ye BF (1994). [N-nitroso-compounds of pickles in the areas with high incidence of digestive cancers and their mutagenic effects]. Zhonghua Yu Fang Yi Xue Za Zhi.

[30] Yamazaki H, Inui Y, Yun CH, Guengerich FP, Shimada T (1992). Cytochrome P450 2E1 and 2A6 enzymes as major catalysts for metabolic activation of N-nitrosodialkylamines and tobacco-related nitrosamines in human liver microsomes. Carcinogenesis.

[31] Fuchs BC, Hoshida Y, Fujii T, Wei L, Yamada S, Lauwers GY, McGinn CM, DePeralta DK, Chen X, Kuroda T, Lanuti M, Schmitt AD, Gupta S (2014). Epidermal growth factor receptor inhibition attenuates liver fibrosis and development of hepatocellular carcinoma. Hepatology.

[32] Kim H, Hong MK, Choi H, Moon HS, Lee HJ (2015). Chemopreventive effects of korean red ginseng extract on rat hepatocarcinogenesis. J Cancer.

[33] Wilson CL, Jurk D, Fullard N, Banks P, Page A, Luli S, Elsharkawy AM, Gieling RG, Chakraborty JB, Fox C, Richardson C, Callaghan K, Blair GE (2015). NFkappaB1 is a suppressor of neutrophil-driven hepatocellular carcinoma. Nat Commun.

[34] Bergmann J, Muller M, Baumann N, Reichert M, Heneweer C, Bolik J, Lucke K, Gruber S, Carambia A, Boretius S, Leuschner I, Becker T, Rabe B (2017). IL-6 trans-signaling is essential for the development of hepatocellular carcinoma in mice. Hepatology.

[35] Zhang H, Gao N, Tian X, Liu T, Fang Y, Zhou J, Wen Q, Xu B, Qi B, Gao J, Li H, Jia L, Qiao H (2015). Content and activity of human liver microsomal protein and prediction of individual hepatic clearance in vivo. Sci Rep.

[36] Ishak KG (1994). Chronic hepatitis: morphology and nomenclature. Mod Pathol.

